# miR-214-3p Mediates Samarium Oxide-Induced Pulmonary Fibrosis by Targeting MAP2K3 via the MAPK Signaling Pathway

**DOI:** 10.3390/toxics14030228

**Published:** 2026-03-08

**Authors:** Ying Sun, Ruixia Ding, Haijing Yin, Teng Ma, Yannan Bi, Sheng Li, Li Wang, Xiaohui Wang

**Affiliations:** 1 School of Public Health, Baotou Medical College, Baotou 014040, China; 15047969392@163.com (Y.S.);; 2 Disease Prevention and Control Center of Wulateqian Banner, Bayannur 014400, China; 3 Basic Medicine and Forensic Science, Baotou Medical College, Baotou 014040, China

**Keywords:** samarium oxide, pulmonary fibrosis, miR-214-3p, MAP2K3, MAPK signaling pathway

## Abstract

**Objective:** Rare-earth elements are extensively employed across diverse industrial sectors, increasingly raising concerns about their potential health hazards in both occupational and environmental contexts. Samarium oxide (Sm_2_O_3_), a routinely processed rare-earth product, reproducibly precipitates pulmonary fibrosis in experimental models, yet the molecular circuitry that transduces its fibrogenic signal remains almost entirely unmapped. This study aims to elucidate the role of miR-214-3p in Sm_2_O_3_-induced pulmonary fibrosis and to investigate its regulatory mechanism at the molecular level. **Methods:** A murine model of pulmonary fibrosis was established via intratracheal instillation of Sm_2_O_3_, and histopathological changes were assessed using hematoxylin and eosin (H&E) and Masson’s trichrome staining. RNA sequencing was performed on lung tissues to identify differentially expressed mRNAs. Leveraging our previously generated miRNA landscape of Sm_2_O_3_-exposed lungs, we subjected the dataset to Gene Ontology and KEGG enrichment analyses, which convergently identified miR-214-3p as the top-ranking candidate regulator of the fibrogenic MAPK axis. The direct targeting of MAP2K3 by miR-214-3p was validated using a dual-luciferase reporter assay. Expression levels of fibrotic markers (α-SMA, Collagen I) and key components of the MAPK signaling pathway (MAP2K3, p-MAPK14, MST1) were quantified in both in vivo and in vitro models using qRT-PCR and Western blotting. Gain- and loss-of-function studies, complemented by rescue assays, were performed in human embryonic lung fibroblasts (HELFs) via transient transfection of miR-214-3p mimics, inhibitors, or MAP2K3-overexpression plasmids. Cell proliferation was evaluated using the EdU assay, and TGF-β1 secretion was measured by ELISA. **Results:** Sm_2_O_3_ exposure induced significant pulmonary fibrosis in mice, accompanied by marked downregulation of miR-214-3p and upregulation of MAP2K3 in lung tissues. Overexpression of miR-214-3p or silencing of MAP2K3 effectively suppressed Sm_2_O_3_-induced fibroblast activation, including reduced cell proliferation, decreased expression of α-SMA and Collagen I, and inhibition of p38 MAPK phosphorylation. Notably, ectopic overexpression of MAP2K3 reversed the protective effects conferred by miR-214-3p, confirming a functional rescue. **Conclusions:** miR-214-3p directly silences MAP2K3, thereby blunting p38 MAPK-driven fibrogenesis after Sm_2_O_3_ exposure. Our data unveil a miR-214-3p–MAP2K3–p38 MAPK axis that constitutes a readily druggable target for rare-earth-element-induced pulmonary fibrosis.

## 1. Introduction

Expanding deployment of rare-earth elements in permanent magnets, catalysts, micro-electronics and emerging energy technologies has elevated them to strategic materials underpinning next-generation industrial development [1]. Consequently, the health hazards linked to occupational exposure to rare-earth elements have recently garnered escalating concern within both the scientific community and the general public [2]. Samarium oxide (Sm_2_O_3_)—a commercially pivotal rare-earth oxide—is readily aerosolized as ultrafine dust during mining, polishing and catalyst operations. Once inhaled, these respirable particles deposit along the distal airways and alveolar epithelium, where their slow clearance fosters chronic bio-persistence and precipitates progressive lung injury [3].

Long-term epidemiological studies and animal experimental evidence have consistently demonstrated that exposure to rare-earth dust can lead to alveolar inflammation and interstitial hyperplasia, indicating potential pulmonary pathological changes [4]. In severe cases, the condition may advance to irreversible pathological alterations, including pulmonary fibrosis (PF), thereby posing a significant threat to the respiratory health of occupational populations [5]. Occupational health examinations revealed a worker engaged in samarium production presenting diffusely distributed ground-glass micronodular opacities in both lungs. Computed tomography (CT) imaging demonstrated a predominance of centrilobular and lymphatic distribution patterns, with denser involvement in the upper and middle lung fields compared to the lower fields. Additionally, faint laminar interstitial thickening parallel to the lobular structures was observed bilaterally. The case was diagnosed as stage II occupational pneumoconiosis [6]. Yin Haijing and colleagues conducted a risk assessment of occupational hazards in a samarium smelting plant in Baotou City. Findings indicated that the total dust concentration in the polishing workshop reached 9.33 mg/m^3^, while that in the raw material workshop was 14.84 mg/m^3^. Both values exceeded China’s occupational exposure limit for rare-earth dust, with a non-compliance rate of 66.67% in dust monitoring. Risk levels for samarium oxide (Sm_2_O_3_) were categorized as extremely high in the production, raw material, and polishing workshops. The operational environment involving Sm_2_O_3_ significantly elevates the risk of pneumoconiosis incidence [7].

The hallmark pathological feature of pulmonary fibrosis is the aberrant activation and excessive proliferation of fibroblasts and myofibroblasts, which play a central role in extracellular matrix deposition and tissue remodeling [8]. This results in the excessive accumulation of extracellular matrix, predominantly consisting of Collagen I, contributing to progressive tissue stiffening and loss of lung function [9]. In this process, transforming growth factor-β1 (TGF-β1) is universally acknowledged as the central mediator of fibrosis, driving the activation and persistence of pro-fibrotic signaling pathways [10]. The p38 mitogen-activated protein kinase (p38 MAPK) signaling pathway represents a central downstream effector mechanism mediating the biological effects, with critical involvement in fibroblast activation and extracellular matrix production [11]. MAP2K3 (also known as MKK3), a key upstream kinase of p38, directly regulates its phosphorylation and activation, serving as a critical initiator of p38 MAPK signaling [12]. The role of MAP2K3 in diverse organ fibrosis models has been well established, demonstrating its involvement in the progression of fibrotic pathologies across multiple tissues [13]. Although the involvement of epigenetic regulation in samarium oxide-induced lung fibrosis has been increasingly recognized, the underlying molecular mechanisms remain incompletely understood [14]. However, the specific molecular mechanisms underlying MAP2K3 function, particularly its epigenetic regulation, remain to be fully elucidated in a systematic manner.

MicroRNAs (miRNAs) are a class of endogenous non-coding small RNAs that play a central role in the post-transcriptional regulation of gene expression by targeting specific mRNAs for degradation or translational repression [15]. MicroRNAs play critical roles in a wide range of pathophysiological processes, including cell proliferation, differentiation, and fibrosis, by fine-tuning gene expression networks [16]. Accumulating evidence indicates that miR-214-3p exhibits cell type- and disease context-dependent expression and function in fibrotic disorders. In cardiac fibrosis, miR-214-3p is highly enriched in cardiac fibroblasts and is significantly upregulated during pathological cardiac remodeling induced by myocardial infarction or heart failure [17]. By contrast, in liver fibrosis, miR-214-3p is predominantly expressed in hepatic stellate cells (HSCs), where its expression is markedly downregulated upon HSC activation and in fibrotic liver tissue. Mechanistically, miR-214-3p exerts anti-fibrotic effects in the liver by directly targeting methyl-CpG-binding protein 2 (MECP2), thereby suppressing HSC proliferation, activation-induced transdifferentiation into myofibroblasts, and excessive extracellular matrix deposition [18]. These findings highlight the dual, context-specific regulatory roles of miR-214-3p in organ-specific fibrosis. However, its role in pulmonary fibrosis remains poorly understood [19]. Notably, the expression profiles and biological functions of miRNAs in rare-earth dust-induced pulmonary fibrosis have not been systematically investigated to date. In the field of fibrotic diseases, there is already successful preclinical evidence for therapeutic strategies aimed at restoring the downregulated miR-214-3p. Zhao et al. (2025) reported in the latest issue of the *Journal of Translational Medicine* that in an alcohol-induced liver fibrosis model, the expression of miR-214-3p was significantly downregulated; the use of adeno-associated virus (AAV)-mediated liver stellate cell-specific overexpression of miR-214-3p could significantly alleviate the degree of liver fibrosis, inhibit stellate cell activation, and reduce extracellular matrix deposition [20].

Accumulating evidence identifies microRNAs (miRNAs) as integral and functionally active regulators within the TGF-β signaling network. Savary et al. first demonstrated that the long non-coding RNA DNM3OS—acting as a direct transcriptional target of TGF-β—serves as a precursor for a pro-fibrotic miRNA cluster, including miR-214-3p, which modulates both canonical (SMAD-dependent) and non-canonical (SMAD-independent) TGF-β signaling pathways through mechanisms of signal amplification and pathway mediation. Subsequent independent studies have consistently validated miR-214-3p as a bona fide TGF-β1-induced “fibromiR”, with functional roles in promoting extracellular matrix deposition and myofibroblast activation across multiple fibrotic contexts, including renal, skeletal muscle, and pulmonary fibrosis [21].

Integrated analysis of RNA-seq data from mouse lung tissues in the present study and proteomic data from blood samples of individuals previously exposed to samarium oxide dust revealed that miR-214-3p expression was significantly downregulated in the lungs of samarium oxide-treated mice [22], whereas its putative target gene MAP2K3 was markedly upregulated. Furthermore, KEGG pathway enrichment analysis indicated significant activation of the MAPK signaling pathway. Based on these findings, we propose the following mechanistic hypothesis: samarium oxide may drive the initiation and progression of pulmonary fibrosis by suppressing miR-214-3p expression, thereby alleviating post-transcriptional repression of MAP2K3, leading to hyperactivation of the p38 MAPK signaling cascade, which in turn promotes TGF-β1 secretion and fibroblast proliferation.

This study employed an integrative approach combining in vivo animal models with in vitro cellular systems to comprehensively investigate the underlying mechanisms [23]. This study aims to systematically validate the regulatory role of the miR-214-3p–MAP2K3–p38 MAPK axis in samarium oxide-induced pulmonary fibrosis, thereby providing a theoretical foundation for elucidating the molecular mechanisms underlying Sm2O3 dust-induced lung pathogenicity and identifying potential therapeutic targets. Moreover, this finding offers mechanistic insight into how environmental particulate matter exposure subverts the evolutionarily conserved TGF-β–miRNA regulatory axis.

## 2. Materials and Methods

### 2.1. Particle Size Characterization of Sm_2_O_3_ Particles

Sm_2_O_3_ powder (purity ≥ 99.9%) was obtained from Baotou Ruixin Rare Earth Smelting Co., Ltd. Baotou, China). Prior to dispersion, the powder was dried at 105 °C for 2 h under ambient atmosphere. A homogeneous suspension was prepared in sterile physiological saline at a nominal dose concentration of 280 mg/kg of body weight (administered volume: 2.0 mL/kg), followed by ultrasonication for 10 min (pulse mode, 30% amplitude) to ensure deagglomeration. Particle size distribution was determined by laser diffraction using a Malvern Mastersizer 3000 instrument. Physiological saline served as the dispersant, and the refractive index was set to 2.05 (a typical value for rare-earth oxides), with the absorption coefficient fixed at 0.1. The volumetric mean diameter (D [3,4]) was 4.45 μm; the cumulative particle size distribution yielded D_10_ = 1.29 μm, D_50_ = 3.82 μm, and D_90_ = 8.52 μm. This aerodynamically relevant size distribution—centered in the fine particulate range (1–10 μm)—supports efficient alveolar deposition following pulmonary exposure and aligns with established size criteria for inhalation toxicology studies of rare-earth oxide particulates.

### 2.2. Animal Model Establishment

Six-week-old male BALB/c mice (18 ± 2 g) were supplied by SPF Biotechnology Co., Ltd. (Beijing, China). Following a 2-week acclimation, they were housed under specific-pathogen-free conditions (22 ± 3 °C, 45–55% relative humidity) with a 12 h light/dark cycle and ad libitum access to irradiated standard chow and sterile reverse-osmosis water. All experimental procedures involving animals were approved by the Laboratory Animal Ethics Committee of Baotou Medical College (Approval No. 027, 2021). Mice were randomly assigned to either the Sm_2_O_3_ exposure group or the control group (*n* = 30 per group). A homogeneous suspension of Sm_2_O_3_ powder was prepared at a nominal concentration of 80 mg/mL in sterile physiological saline. Mice in the exposure group received a single intratracheal instillation of 0.1 mL of the Sm_2_O_3_ suspension, while control animals received an equivalent volume of normal saline [24]. Thirty-five days after exposure, the mice were euthanized under anesthesia.

This study utilized a single-dose, non-invasive intratracheal instillation method—a well-established and widely accepted modeling approach for rare-earth-induced pulmonary fibrosis. The procedure was performed strictly according to the standardized protocol described by Zhao et al. (2021) [3] for Sm_2_O_3_ exposure: following anesthesia, mice were positioned supine and secured; the tongue was gently retracted to visualize the glottis; at the peak of glottic opening, a blunted gavage needle was carefully advanced into the trachea to a depth of 1.0–1.5 cm; successful placement was confirmed by gentle aspiration (absence of resistance or fluid return), followed by slow, bolus delivery of the Sm_2_O_3_ suspension; post instillation, animals were maintained in a head-elevated position until full recovery of consciousness.

### 2.3. HE and Masson Staining

Lung tissues from both mouse groups were harvested and fixed in 4% paraformaldehyde for 24 h, followed by graded ethanol dehydration, xylene clearing, and routine paraffin embedding [25]; slice thickness is 4 μm.

HE staining: Following deparaffinization and rehydration, tissue sections were stained with hematoxylin for 6 min, rinsed under running tap water for 10 min to promote blueing, differentiated in acid alcohol for 3 min [26], and counterstained with eosin for 3 min. Subsequently, sections were dehydrated through a graded ethanol series, cleared in xylene, and mounted with neutral balsam for long-term preservation.

Semi-quantitative assessment of pulmonary fibrosis: The modified Ashcroft scoring system (0–8 points) was used. Five non-overlapping fields (200×) were randomly selected from each mouse and independently evaluated by two blinded assessors. The scoring criteria were based on Hübner et al. [27], ranging from 0 points (normal) to 8 points (fibrotic tissue throughout the field). The inter-rater agreement between the two observers was good.

Masson staining: Following deparaffinization and rehydration, tissue sections were stained with Weigert’s hematoxylin for 7 min, rinsed under running tap water for 25 min to achieve complete blueing, then stained with Masson’s acid fuchsin solution for 7 min and briefly washed in 2% glacial acetic acid [28]. Sections were subsequently immersed in 1% phosphomolybdic acid aqueous solution for 5 min to selectively differentiate collagen components, followed by a brief rinse in 0.2% glacial acetic acid. Finally, sections were counterstained with aniline blue for 5 min, dehydrated through a graded ethanol series, cleared in xylene, and mounted with neutral balsam.

Quantification of Masson’s trichrome-stained collagen area: Standardized image analysis was performed using ImageJ Fiji software with the Colour Deconvolution plugin. The specific steps were as follows: ① import 24-bit RGB images into ImageJ; ② Plugins → Colour Deconvolution → ‘Masson Trichrome’ preset vector to separate the blue channel; ③ threshold segmentation to generate a binary image, and calculate the percentage of blue collagen area in the total area of the lung parenchyma ROI (Area%); and ④ the average value of 25 fields (5 sections × 5 fields) from each mouse was used for statistical analysis [29].

### 2.4. RNA Sequencing and Bioinformatics Analysis

Total RNA was extracted from lung tissue samples of three mice in both the control group and the Sm_2_O_3_-treated group using TRIzol reagent [30]. RNA sequencing (Shanghai Lianchuang Biomedical Technology Co., Ltd., Shanghai, China). Differentially expressed miRNAs (DEmiRs) and mRNAs (DEmRNAs) were identified using a threshold of |log_2_ fold change| ≥ 2 and adjusted *p*-value < 0.01. The mouse RNA-seq data were subsequently integrated with the research group’s prior proteomic profiling results from blood samples of samarium oxide dust-exposed individuals for comparative analysis.

Potential target genes of miR-214-3p were predicted using TargetScan and miRDB databases. The overlapping targets from both databases were intersected with differentially expressed mRNAs (DEmRNAs) to identify candidate target genes [31]. KEGG pathway enrichment analysis of the candidate target genes was performed using the Metascape database to identify key signaling pathways associated with pulmonary fibrosis [32].

### 2.5. Cell Culture

Human embryonic lung fibroblasts (HELFs) (Shanghai Sunshine Biological Co., Ltd., Shanghai, China) and were cultured in DMEM high-glucose medium (Meilun Biological, Shanghai, China) [33], 10% fetal bovine serum (Meilun Bio, Shanghai, China) and 1% penicillin–streptomycin double antibody (Meilun Bio, Shanghai, China) were added to the culture medium. The cells were routinely cultured in a 37 °C, 5% CO_2_ incubator (Waltham, MA, USA).

### 2.6. Treatment of Cells with Sm_2_O_3_

For cellular assays, a stock solution was prepared by dissolving 5 mg of Sm_2_O_3_ in 5 mL of sterile phosphate-buffered saline (PBS) [34]. Subsequent serial dilutions were performed in RPMI-1640 medium to yield final working concentrations of 0, 3.125, 6.25, and 12.5 μg/mL.

### 2.7. RNA Extraction and Quantitative Real-Time PCR (qRT-PCR) Analysis

Total RNA was extracted from lung tissue using the TransZol reagent (Transgen Biotech, Beijing, China), and its concentration and quality were assessed [35]. Reverse transcribe total RNA using the transcription system (Vazyme Biotech Co., Nanjing, China) [36]. Reverse transcribe at 55–60 °C for 10 min, and then store at −80 °C for long-term use. Detect the gene expression level using a qRT-PCR kit (Vazyme Biotech Co., Nanjing, China) on a Light Cyker96 instrument (Roche, Basel, Switzerland) [37]. The amplification conditions were as follows: initial denaturation at 95 °C for 30 s, followed by 40 cycles of denaturation at 95 °C for 10 s, and combined annealing/extension at 60 °C for 30 s. A final melt curve analysis was performed with the following steps: 95 °C for 15 s, 60 °C for 60 s, and 95 °C for 15 s. Gene expression levels were quantified using the 2^−ΔΔCT^ method with three biological replicates. The primer sequences used in this study are listed in Table 1.

### 2.8. Western Blot for Protein Expression Detection

Lung tissue or cells were lysed in RIPA buffer supplemented with protease and phosphatase inhibitors, followed by incubation on ice for 30 min [38]. The samples were then centrifuged at 12,000× *g* for 15 min at 4 °C, and the resulting supernatant was collected for protein quantification using the BCA assay [39]. Protein concentration was determined, and 50 μg of protein samples were subjected to SDS-PAGE electrophoresis, followed by wet transfer onto PVDF membranes. Membranes were blocked with 5% skim milk at room temperature for 2 h. Primary antibodies against α-SMA (rabbit, 1:1000), Collagen I (1:1000), MAP2K3 (1:1000), MAPK14 (1:1000), MST1 (1:1000), p-p38 (1:1000), and GAPDH (1:5000) were applied and incubated overnight at 4 °C. After three washes with TBST, membranes were incubated with HRP-conjugated secondary antibodies (1:5000) for 1 h at room temperature. Protein bands were visualized using an ECL chemiluminescence kit and imaged with a gel imaging system [40]; band intensities were quantified using ImageJ software, and the relative expression level of the target protein was normalized to the internal reference GAPDH by calculating the ratio of the target protein’s band intensity to that of GAPDH [41].

### 2.9. Dual-Luciferase Reporter Gene Assay

According to TargetScan prediction [42], the binding site between miR-214-3p and the 3′UTR of MAP2K3 was predicted using TargetScan, and wild-type (WT) and mutant (MUT) MAP2K3 3′UTR luciferase reporter vectors (pmirGLO-MAP2K3-WT and pmirGLO-MAP2K3-MUT) (Shanghai GenePharma Co., Ltd., Shanghai, China). Human embryonic lung fibroblasts (HELFs) were seeded into 24-well plates and transfected at 70–80% confluence. Following the manufacturer’s protocol for Lipofectamine 3000, an miR-214-3p mimic or a negative-control (NC) mimic was co-transfected with either pmirGLO-MAP2K3-WT or pmirGLO-MAP2K3-MUT. After 48 h, cells were harvested and lysed with lysis buffer from the dual-luciferase reporter assay kit. Firefly and Renilla luciferase activities were measured sequentially, and relative luciferase activity was calculated as the ratio of firefly to Renilla luminescence [43].

### 2.10. Cell Transfection and Functional Experiments

HELF cells were seeded in 6-well plates and transfected upon reaching 70–80% confluence. The experiment included the following groups: the control group (untreated), Sm_2_O_3_ group (treated with 100 μg/mL Sm_2_O_3_), Sm_2_O_3_ + NC mimic group, Sm_2_O_3_ + miR-214-3p mimic group, Sm_2_O_3_ + si-NC group, Sm_2_O_3_ + si-MAP2K3 group, Sm_2_O_3_ + miR-214-3p mimic + pcDNA3.1 group, and Sm_2_O_3_ + miR-214-3p mimic + pcDNA3.1-MAP2K3 group. Transfections were carried out according to the manufacturer’s instructions for the transfection reagent. After 48 h, the cells were treated with 100 μg/mL Sm_2_O_3_ for an additional 24 h prior to downstream analyses.

### 2.11. EdU Cell Proliferation Assay

Transfected and treated cells were seeded into 96-well plates and incubated with a 50 μmol/L EdU working solution at 37 °C for 2 h. After PBS washing, the cells were fixed with 4% paraformaldehyde for 30 min, permeabilized with 0.5% Triton X-100 for 10 min, then incubated with Click reaction mixture in the dark for 30 min. Nuclei were counterstained with Hoechst 33,342 for 10 min [44]. Fluorescence microscopy was used to capture images from five randomly selected fields, and the number of EdU-positive cells (red) and total cells (blue) were counted. The cell proliferation rate was calculated as the percentage of EdU-positive cells relative to total cells (EdU-positive cell count/total cell count × 100%).

### 2.12. ELISA Was Used to Detect the Level of TGF-β1

Mouse lung tissues were harvested and homogenized in physiological saline to prepare 10% (*w*/*v*) tissue homogenates. The homogenates were centrifuged at 3000× *g* for 10 min at 4 °C, and the supernatants were collected. Cell culture supernatants were also collected [45]. ELISA was performed according to the manufacturer’s instructions. Absorbance at 450 nm was measured using a microplate reader, and TGF-β1 concentrations were determined based on the standard curve.

### 2.13. Statistical Analysis

Data analysis was conducted using SPSS 26.0 software [46]. GraphPad Prism 8.0 software was used to draw the charts [47]. Measurement data are presented as mean ± standard deviation ($\bar{x}$ ± s). Comparisons between two groups were performed using the independent samples *t*-test, while comparisons among multiple groups were conducted using one-way analysis of variance (ANOVA). Post hoc pairwise comparisons were carried out using the LSD-t test. A value of *p* < 0.05 was considered statistically significant.

## 3. Result

### 3.1. Sm_2_O_3_ Can Induce Pulmonary Fibrosis in Mice

**Figure 1:**
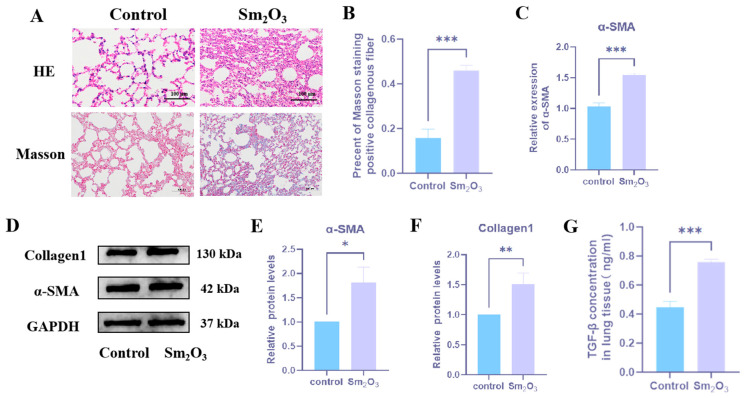
Effects of Sm_2_O_3_ exposure on pulmonary fibrosis in mice. (**A**) HE and Masson staining of mouse lung tissue; (**B**) positive area ratio of Masson staining (means ± SD, *n* = 6) *** *p* < 0.001; (**C**) α-SMA mRNA level in mouse lung tissue (means ± SD, *n* = 6); (**D**–**F**) protein expression levels of α-SMA and Collagen 1 (means ± SD, *n* = 6); and (**G**) TGF-β secretion level (means ± SD, *n* = 6). Compared with the control group: * *p* < 0.05, ** *p* < 0.01, *** *p* < 0.001.

The effects of Sm_2_O_3_ on mouse lung tissue were assessed by histopathological analysis. HE staining revealed well-preserved lung architecture and intact alveolar structures in the control group, with no evident inflammatory cell infiltration. In contrast, Sm_2_O_3_-treated mice exhibited disrupted alveolar architecture, markedly thickened septa, and extensive inflammatory cell infiltration (Figure 1A). Masson trichrome staining further demonstrated a substantial increase in blue-stained collagen fiber deposition in the lungs of Sm_2_O_3_-treated mice compared to controls (*p* < 0.01; Figure 1B,C), indicating significant extracellular matrix accumulation.

At the molecular level, qRT-PCR analysis revealed significantly higher mRNA expression of the fibrosis markerα-SMA in the lung tissue of Sm_2_O_3_-treated mice compared to controls. Western blot analysis further demonstrated elevated protein levels of both α-SMA and Collagen I in the Sm_2_O_3_ group relative to the control group (*p* < 0.05; Figure 1D–F). Moreover, ELISA analysis of lung tissue homogenates showed a significant increase in the concentration of TGF-β1 in the Sm_2_O_3_ group (*p* < 0.01; Figure 1G), indicating enhanced profibrotic signaling.

### 3.2. miR-214-3p Is Downregulated in Sm_2_O_3_-Induced Pulmonary Fibrosis and Targets MAP2K3 for Regulation

**Figure 2:**
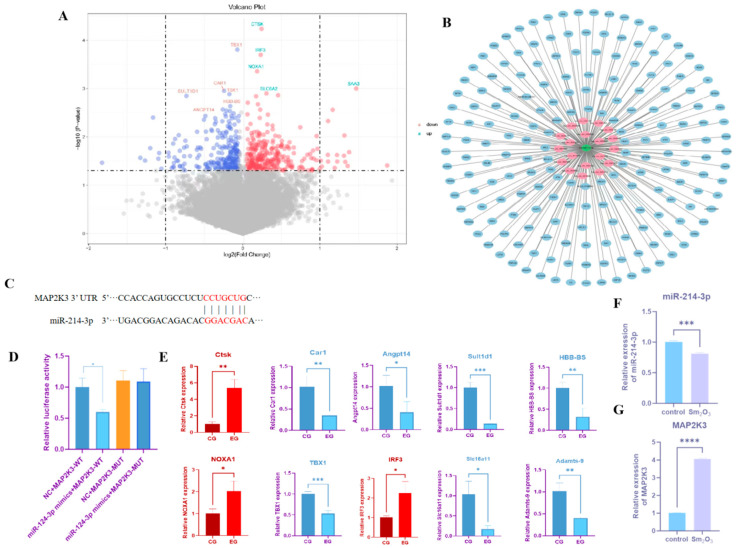
Transcriptome sequencing results of the Sm_2_O_3_ exposure group. (**A**) Volcano plot of differentially expressed genes in mouse lung tissue RNA-seq; (**B**) PPI network of differentially expressed genes in mouse RNA-seq sequencing combined with proteomics of blood from samarium oxide dust exposed individuals; pink indicates genes with a declining trend, while blue indicates genes with an ascending trend; (**C**) binding site of miR-214-3P and MAP2K3; (**D**) dual-luciferase assay results (means ± SD, *n* = 6); (**E**) qRT-PCR detection results of differentially expressed genes in mouse lung tissue RNA-seq; and (**F**,**G**) the qRT-PCR expression levels of miR-214-3p and MAP2K3 in the lung tissues of mice (means ± SD, *n* = 6). Compared with the control group: * *p* < 0.05, ** *p* < 0.01, *** *p* < 0.001, **** *p* < 0.0001.

Differentially expressed genes were identified from mouse RNA-seq data, and identify the significantly differentially expressed genes through volcano plots (Figure 2A). To identify key molecular determinants of Sm_2_O_3_-induced pulmonary fibrosis, we integrated RNA-seq data from lung tissues of Sm_2_O_3_-exposed mice with proteomic profiles from peripheral blood of occupationally exposed individuals (previously collected by our group). Orthologous human–mouse genes were identified, and cross-platform differential expression analysis yielded at both transcriptomic and proteomic levels. This set was subjected to protein–protein interaction (PPI) network analysis using STRING, revealing MAP2K3 as a topologically central node—exhibiting high-degree centrality and robust functional associations with p38 MAPK pathway components and canonical fibrotic effectors. These findings implicate MAP2K3 as a putative signaling nexus in Sm_2_O_3_-associated fibrogenesis. Subsequent bioinformatic prediction using TargetScan and miRDB identified miR-214-3p as a high-confidence, evolutionarily conserved regulator of MAP2K3, with an 8-mer seed match in its 3′UTR. Critically, miR-214-3p was significantly downregulated in the murine RNA-seq dataset and showed strong inverse correlation with MAP2K3 mRNA abundance. Collectively, integrative multi-omics and target prediction analyses nominate the miR-214-3p–MAP2K3 regulatory axis as the leading mechanistic candidate for experimental validation (Figure 2B,C). Dual-luciferase reporter assays further confirmed that overexpression of miR-214-3p significantly suppressed the luciferase activity of the wild-type MAP2K3 3′-UTR reporter (*p* < 0.01), but not that of the mutant construct harboring a disrupted binding site (Figure 2D), demonstrating that MAP2K3 is a direct functional target of miR-214-3p. Significantly differentially expressed genes were identified through volcano plots and verified by qPCR. The qPCR results were consistent with the transcriptome sequencing results, confirming the authenticity of the mouse RNA-seq (Figure 2E). The expression levels of miR-214-3p and MAP2K3 were assessed by qRT-PCR. Compared with the control group, miR-214-3p expression in the lung tissues of Sm_2_O_3_-treated mice was significantly downregulated, whereas MAP2K3 mRNA expression was markedly upregulated (*p* < 0.05), consistent with the sequencing data (Figure 2F,G).

### 3.3. Sm_2_O_3_ Promotes the Process of Pulmonary Fibrosis by Activating the MAPK Signaling Pathway Through the miR-214-3p–MAP2K3 Axis

**Figure 3:**
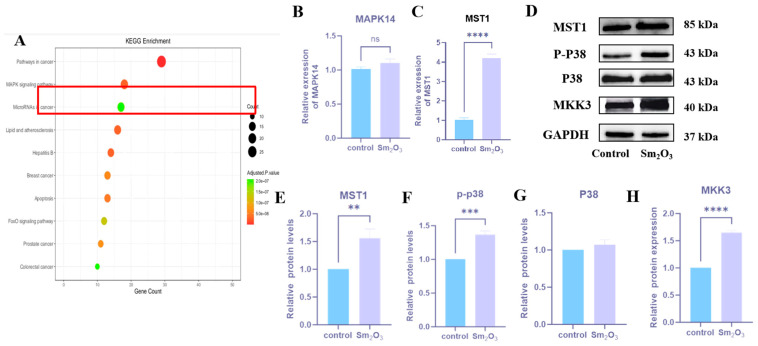
Detection of MAPK signaling pathway-related factors in mouse lung tissue. (**A**) KEGG signaling pathway analysis of differentially expressed genes; (**B**,**C**) qRT-PCR expression levels of MAPK signaling pathway-related factors MAPK14 and MST1 in mouse lung tissue (means ± SD, *n* = 6). Compared with the control group: **** *p* < 0.0001. (**D**–**H**) Protein expression levels of MAPK signaling pathway-related factors MKK3 (MAP2K3), P38 (MAPK14), p-P38, and MST1 in mouse lung tissue (means ± SD, *n* = 6). Compared with the control group: ** *p* < 0.01, *** *p* < 0.001, **** *p* < 0.0001.

KEGG analysis of the sequencing data revealed significant enrichment of the MAPK signaling pathway in the Sm_2_O_3_ treatment group (*p* < 0.01), indicating its potential involvement in pulmonary fibrosis (Figure 3A). The expression levels of key pathway molecules were examined using qRT-PCR and Western blot. Compared with the control group, MST1 mRNA expression was significantly upregulated in the lung tissues of Sm_2_O_3_-treated mice (*p* < 0.05), whereas no significant differences were observed in the mRNA levels of total MAPK14 across groups (*p* > 0.05) (Figure 3B,C). At the protein level, MAP2K3 and MST1 were both significantly elevated in the Sm_2_O_3_ group (*p* < 0.05), and phosphorylated p38 MAPK (p-MAPK14) expression was also markedly increased (*p* < 0.05), while total MAPK14 protein levels remained unchanged (*p* > 0.05) (Figure 3D–H).

### 3.4. Sm_2_O_3_ Activates the MAPK Signaling Pathway and Promotes the Fibrotic Response of HELF Cells

This study investigated the effects of different concentrations of Sm_2_O_3_ exposure on the fibrotic phenotype and MAPK signaling pathway of HELF. After the cells were treated with 0, 3.125, 6.25 and 12.5 μg/mL Sm_2_O_3_ for 24 h, respectively, the expression of related molecular markers was detected.

**Figure 4:**
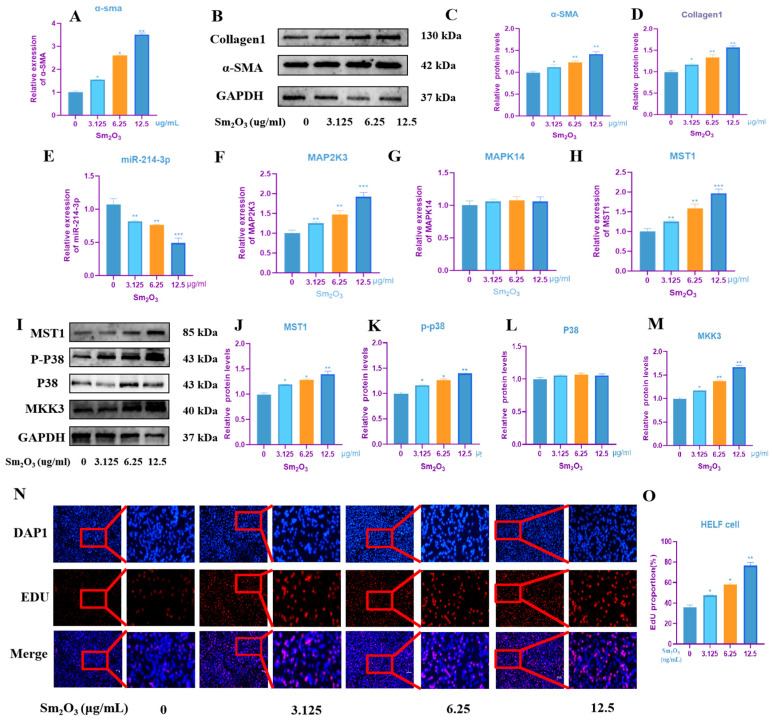
Effects of Sm_2_O_3_ exposure on HELF human embryonic lung fibroblasts and detection of MAPK signaling pathway-related factors. (**A**) α-SMA mRNA level in HELF cells (means ± SD, *n* = 3); (**B**–**D**) protein expression levels of α-SMA and Collagen 1 (means ± SD, *n* = 3); (**E**–**H**) qRT-PCR expression levels of miR-214-3p and MAPK signaling pathway-related factors MAP2K3, MAPK14, and MST1 in HELF cells (means ± SD, *n* = 3); (**I**–**M**) protein expression levels of MAPK signaling pathway-related factors MKK3 (MAP2K3), P38 (MAPK14), p-P38, and MST1 in HELF cells (means ± SD, *n* = 3); and (**N**,**O**) the EDU/DAPI ratio indicates that Sm_2_O_3_ exposure promotes the proliferation of HELF cells (means ± SD, *n* = 3). Compared with the control group: * *p* < 0.05, ** *p* < 0.01, *** *p* < 0.001.

qRT-PCR and Western blot analyses revealed that, in a dose-dependent manner, the mRNA and protein expression levels of the fibrosis marker α-SMA, as well as the protein level of Collagen I, were significantly elevated in HELF cells compared to the control group (*p* < 0.05; Figure 4A–D). qRT-PCR results further demonstrated that increasing Sm_2_O_3_ concentrations led to a significant downregulation of miR-214-3p expression and concurrent upregulation of MAP2K3 and MST1 mRNA levels in human embryonic lung fibroblasts (*p* < 0.05) (Figure 4E–H). Consistently, Western blot analysis showed that MAP2K3 and MST1 protein levels were significantly increased in Sm_2_O_3_-treated groups, along with a marked increase in p-MAPK14 (*p* < 0.05), while total MAPK14 levels remained unchanged (Figure 4I–M). Furthermore, EdU proliferation assays confirmed that Sm_2_O_3_ treatment significantly enhanced the proliferative activity of HELF cells in a dose-dependent manner (*p* < 0.01; Figure 4N,O).

### 3.5. Overexpression of miR-214-3p Inhibits the Fibrotic Phenotype of HELF Cells and the Activation of the MAPK Signaling Pathway

**Figure 5:**
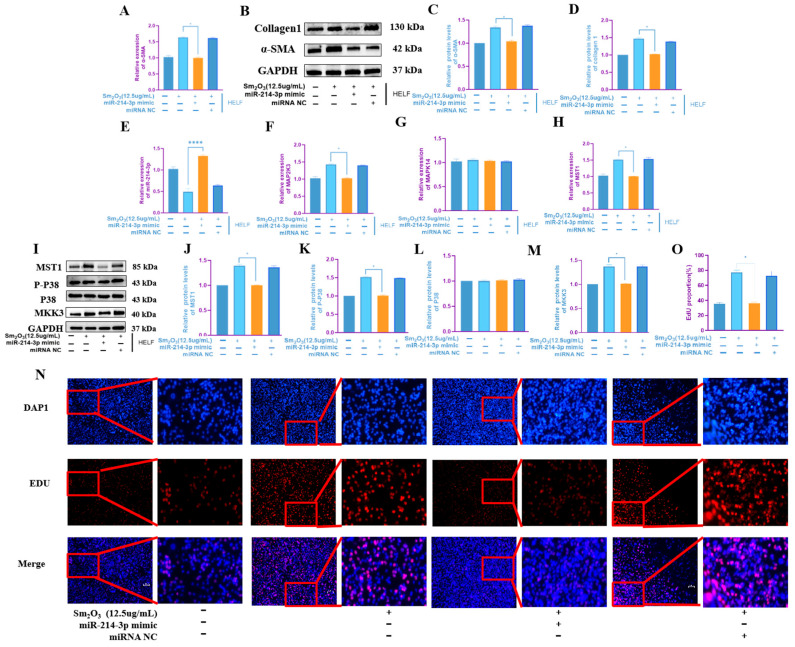
Effects of overexpressing miR-214-3p on HELF human embryonic lung fibroblasts. (**A**) α-SMA mRNA levels in HELF cells after overexpressing miR-214-3p (means ± SD, *n* = 3); (**B**–**D**) protein expression levels of α-SMA and Collagen 1 (means ± SD, *n* = 3); (**E**–**H**) qRT-PCR expression levels of miR-214-3p and MAPK signaling pathway-related factors MAP2K3, MAPK14, and MST1 in HELF cells after overexpressing miR-214-3p (means ± SD, *n* = 3); (**I**–**M**) protein expression levels of MAPK signaling pathway-related factors MKK3 (MAP2K3), P38 (MAPK14), p-P38, and MST1 in HELF cells (means ± SD, *n* = 3); and (**N**,**O**) the EDU/DAPI ratio indicates that the proliferation of HELF cells is inhibited after overexpressing miR-214-3p (means ± SD, *n* = 3). Compared with the high-dose group: * *p* < 0.05, **** *p* < 0.0001.

HELF cells were transfected with miR-214-3p mimic to achieve overexpression and then exposed to 12.5 μg/mL Sm_2_O_3_ for 24 h. qRT-PCR and Western blot analyses demonstrated that miR-214-3p overexpression significantly suppressed the mRNA and protein expression of the fibrotic marker α-SMA, as well as the protein level of Collagen I, compared to the Sm_2_O_3_-only group (*p* < 0.05; Figure 5A–D). The qRT-PCR results confirmed successful miR-214-3p upregulation and revealed significant downregulation of MAP2K3 and MST1 mRNA levels (*p* < 0.05), while no significant change was observed in other targets (*p* > 0.05) (Figure 5E–H). At the protein level, Western blot analysis showed that miR-214-3p overexpression markedly reduced the expression of MAP2K3, MST1, and phosphorylated p38 MAPK, indicating suppression of MAPK pathway activation (Figure 5I–M). Furthermore, EdU proliferation assays indicated that miR-214-3p overexpression significantly inhibited HELF cell proliferative activity (*p* < 0.01; Figure 5N,O).

### 3.6. Knockdown of MAP2K3 Mimics the Anti-Fibrotic Effect of miR-214-3p Overexpression

**Figure 6:**
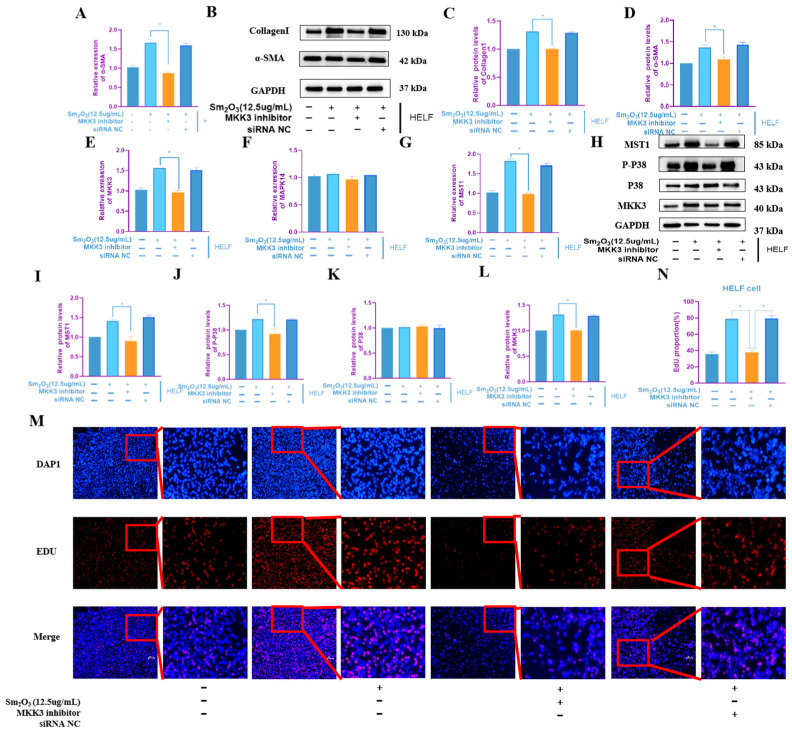
Effects of MKK3 knockdown on HELF human embryonic lung fibroblasts. (**A**) α-SMA mRNA levels in HELF cells after MKK3 knockdown (means ± SD, *n* = 3); (**B**–**D**) protein expression levels of α-SMA and Collagen 1 (means ± SD, *n* = 3); (**E**–**G**) qRT-PCR expression levels of MAPK signaling pathway-related factors MAP2K3, MAPK14, and MST1 in HELF cells after MKK3 knockdown (means ± SD, *n* = 3); (**H**–**L**) protein expression levels of MAPK signaling pathway-related factors MKK3 (MAP2K3), P38 (MAPK14), p-P38, and MST1 in HELF cells after MKK3 knockdown (means ± SD, *n* = 3); and (**M**,**N**) EDU/DAPI ratio showing that the proliferation of HELF cells was inhibited after MKK3 knockdown (means ± SD, *n* = 3). Compared with the high-dose group: * *p* < 0.05.

HELF cells were transfected with RNA interference constructs to knock down MAP2K3 expression and subsequently exposed to 12.5 μg/mL Sm_2_O_3_ for 24 h. qRT-PCR and Western blot analyses revealed that MAP2K3 knockdown significantly reduced the mRNA and protein expression levels of the fibrotic marker α-SMA, as well as the protein level of Collagen I, compared to the Sm_2_O_3_-only control group (*p* < 0.05; Figure 6A–D). qRT-PCR results confirmed efficient silencing of MAP2K3 and showed a significant downregulation of MST1 mRNA (*p* < 0.05) (Figure 6E–G). At the protein level, Western blot analysis demonstrated that MAP2K3 knockdown markedly decreased the expression of MAP2K3, p-MAPK14, and MST1, indicating suppression of downstream MAPK signaling (Figure 6H–L). Furthermore, EdU proliferation assays indicated that silencing MAP2K3 significantly inhibited HELF cell proliferative activity (*p* < 0.01; Figure 6M,N).

### 3.7. Overexpression of MAP2K3 Reverses the Anti-Fibrotic Effect Mediated by miR-214-3p

**Figure 7:**
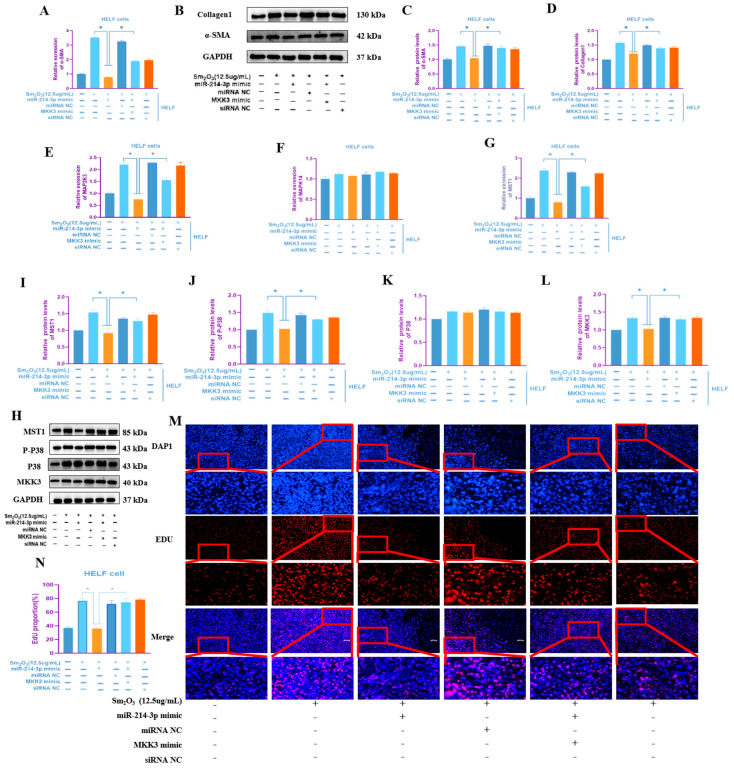
Overexpression of MKK3 Reverses the Effects of Overexpressed miR-214-3p on HELF Human Embryonic Lung Fibroblasts. (**A**) Overexpression of MKK3 Reverses the effects of overexpressed miR-214-3p on α-SMA mRNA levels in HELF cells (means ± SD, *n* = 3); (**B**–**D**) overexpression of MKK3 reverses the effects of overexpressed miR-214-3p on protein expression levels of α-SMA and Collagen 1 (means ± SD, *n* = 3); (**E**–**G**) qRT-PCR expression levels of MAPK signaling pathway-related factors MAP2K3, MAPK14, and MST1 in overexpression of MKK3 reversing overexpression of miR-214-3p (means ± SD, *n* = 3); (**H**–**L**) protein expression levels of MKK3 (MAP2K3), P38 (MAPK14), p-P38, and MST1 in overexpression of MKK3 reversing overexpression of miR-214-3p (means ± SD, *n* = 3); and (**M**,**N**) the EDU/DAPI ratio shows that overexpression of MKK3 reverses the inhibitory effect of overexpressed miR-214-3p on the proliferation of HELF cells (means ± SD, *n* = 3). Compared with the miR-214-3p mimic group: * *p* < 0.05.

HELF cells overexpressing miR-214-3p were co-transfected with a MAP2K3 overexpression plasmid. qRT-PCR and Western blot analyses demonstrated that MAP2K3 overexpression reversed the downregulation of the fibrotic marker α-SMA at both mRNA and protein levels, as well as the reduction in Collagen I protein expression, induced by miR-214-3p overexpression (*p* < 0.05; Figure 7A–D). qRT-PCR results confirmed that MAP2K3 overexpression significantly upregulated the mRNA expression of both MAP2K3 and MST1 (*p* < 0.05) (Figure 7E–G). Western blot analysis showed that, compared to cells transfected with the miR-214-3p mimic alone, the co-transfection group exhibited significantly increased protein levels of MAP2K3, p-MAPK14, and MST1 (Figure 7H–L). Furthermore, EdU proliferation assays revealed that MAP2K3 overexpression effectively rescued the suppression of HELF cell proliferation mediated by miR-214-3p (*p* < 0.01; Figure 7M,N). In summary, the findings of this study demonstrate that during pulmonary fibrosis progression, Sm_2_O_3_ acts on fibroblasts by downregulating miR-214-3p expression, thereby promoting the expression of its downstream target MAP2K3 mRNA and subsequently activating the p-P38–MST1 signaling axis. This activation enhances fibroblast proliferation and exacerbates the progression of pulmonary fibrosis.

## 4. Discussion

Pulmonary fibrosis is a severe lung pathology characterized by progressive extracellular matrix deposition and impaired respiratory function. Its development is closely associated with exposure to various environmental noxious agents, among which rare-earth oxides have emerged as significant contributors to pulmonary injury and fibrogenesis. In recent years, growing evidence has highlighted the critical role of epigenetic regulation in the pathogenesis of pulmonary fibrosis [48]. Non-coding RNAs, including miRNAs, have emerged as promising therapeutic targets for intervention. In this study, an intratracheal exposure mouse model was established using Sm_2_O_3_, which exhibited alveolar structural disruption, excessive collagen deposition, and significantly upregulated expression of key fibrotic markers, including α-SMA, type I collagen, and TGF-β1. These findings further confirm the pathogenic role of this class of rare-earth oxides in the development of pulmonary fibrosis. Compared with the common inducers of pulmonary fibrosis (such as bleomycin and silica), the pathogenic mechanism of rare-earth oxides is still in its infancy. Epidemiological investigations have shown that rare-earth industry workers have an increased risk of respiratory diseases [49], but the mechanistic evidence is mostly limited to oxidative stress. This study is the first to link rare-earth exposure to specific miRNA regulatory networks using in vitro and in vivo models, providing new molecular clues to explain the chronic pathogenicity of rare-earth dust.

At the mechanism level, miRNA has been proven to play a key role in the regulatory network of pulmonary fibrosis [50]. The expression of miR-214-3p was significantly decreased in Sm_2_O_3_-treated lung tissues, which is highly consistent with the expression profile observed in tissues from patients with idiopathic pulmonary fibrosis (IPF) and in bleomycin- and silica-induced animal models [51]. These results suggest that downregulation of miR-214-3p may be a conserved fibrosis-promoting event independent of pathogenic factors. However, unlike the upregulation of miR-214-3p observed in some tumor-associated fibrosis, the present results tend to support its anti-fibrotic role in fibroproliferative diseases [52]. These findings suggest that miR-214-3p may serve as an evolutionarily conserved negative regulator involved in the common pathogenic pathway underlying fibrosis induced by diverse etiological factors. Further cellular experiments demonstrate that overexpression of miR-214-3p effectively suppresses fibroblast activation, consistent with its established anti-fibrotic function. This enhances its potential value as a therapeutic target.

**Figure 8:**
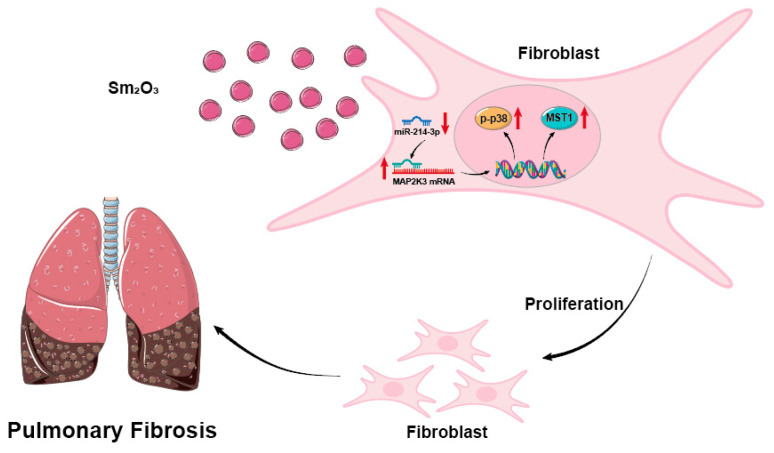
miR-214-3p regulates the MAP2K3–MAPK axis and participates in Sm_2_O_3_-induced pulmonary fibrosis.

To elucidate its downstream mechanism, bioinformatics analysis combined with experimental validation identified MAP2K3 as a direct target of miR-214-3p. As a key kinase in the MAPK signaling pathway, MAP2K3 activates p38 MAPK, thereby promoting the expression of pro-fibrotic factors such as TGF-β1. In Sm_2_O_3_-induced fibrotic lung tissues, miR-214-3p and MAP2K3 exhibit a significant negative correlation in expression. More importantly, overexpression of miR-214-3p was shown to suppress activation of the MAP2K3–p38 MAPK axis, reduce levels of downstream fibrotic markers, and inhibit fibroblast proliferation. These results echo the conclusion that other mirnas (such as miR-21 and let-7) regulate fibrosis through the MAPK pathway in previous studies [53], and MAP2K3, as a direct kinase upstream of p38, has previously been reported to be non-transcriptional, regulated by TGF-β in pulmonary fibrosis [54]. The miR-214-3p–MAP2K3 axis revealed in this study provides new insights into the post-transcriptional regulation of this pathway, and this study is characterized by the specific regulation of Mir-214-3p–MAP2K3–p38 axis in the context of rare-earth-induced pulmonary fibrosis [55], but distinct contribution of this study lies in uncovering the specific regulatory function of the miR-214-3p–MAP2K3–p38 axis in the context of rare-earth-induced pulmonary fibrosis, thereby advancing our understanding of the pathogenic mechanisms underlying environmental toxicant exposure (Figure 8).

Compared to previous studies, the strength of this work lies in the systematic elucidation of a comprehensive mechanistic cascade—from environmental exposure to micro-regulatory molecules, through signaling pathways, and ultimately to cellular phenotypes—providing novel insights into the molecular basis of rare-earth-associated pulmonary fibrosis. Nevertheless, this study has certain limitations. Notably, in vivo validation has not yet been performed using miR-214-3p gene knockout or conditional overexpression animal models, which limits the ability to fully evaluate the regulatory capacity of this miRNA under integrated physiological and pathological conditions. This represents a relative limitation compared to studies that have employed genetically modified models. A single-dose, non-invasive intratracheal instillation model using a high concentration of Sm_2_O_3_ was employed. This experimental paradigm differs substantially from the chronic, low-dose, inhalation-based occupational exposure experienced by workers in the rare-earth industry. While the present study centers on MAP2K3 as a key downstream effector, it is important to acknowledge that miR-214-3p regulates a broad and functionally heterogeneous network of targets—many of which are implicated in fibrotic pathways. Comprehensive validation of these additional candidates was beyond the scope of this investigation; thus, their functional relevance remains to be determined and represents a logical focus for future mechanistic studies. Although miR-214-3p expression was significantly downregulated in whole-lung homogenates from Sm_2_O_3_-exposed mice, subsequent functional validation—including loss- and gain-of-function assays—was conducted specifically in primary lung fibroblasts to dissect cell type-specific regulatory mechanisms [56].

Despite these limitations, our study has important translational implications and potential applications. Firstly, at the biological monitoring level, the expression level of miR-214-3p in peripheral blood has been explored as a biomarker in a variety of diseases. Combined with the results of this study, its early warning value in the physical screening of rare-earth workers may be explored in the future. Secondly, at the level of therapeutic intervention, mimics of miR-214-3p or small-molecule inhibitors of MAP2K3 (such as p38 inhibitors, which have been clinically explored) may provide “old drugs for new use” or precise intervention strategies for rare-earth-induced pulmonary fibrosis. In addition, the epigenetic regulatory mechanism revealed in this study suggests that intervention of epigenetic changes caused by environmental exposure may have more fundamental preventive significance than simple anti-inflammatory or anti-oxidation effects, which provides a new theoretical basis for the development of occupational protection strategies in the rare-earth industry.

## 5. Conclusions

This study demonstrates that Sm_2_O_3_ exposure downregulates miR-214-3p expression, thereby relieving its targeted repression of the MAP2K3 gene, leading to increased MAP2K3 expression and subsequent activation of the p38 MAPK signaling pathway. This cascade promotes fibroblast proliferation and ultimately drives the development of pulmonary fibrosis. The miR-214-3p–MAP2K3–p38 MAPK axis plays a critical regulatory role in Sm_2_O_3_-induced pulmonary fibrosis, providing a novel molecular mechanism underlying the disease pathogenesis. Furthermore, these findings highlight miR-214-3p and MAP2K3 as promising therapeutic targets for the prevention and treatment of rare-earth element-associated pulmonary fibrosis.

## Figures and Tables

**Table 1:** Primer Sequences.

Genes	Sequences
α-SMA	F:ATGACCCAGATTATGTTTGAGACCTTC R:TCTCCAGAGTCCAGCACAATACC
MAP2K3	F:CCCCAGTCCAAAGAGAGGCT R:GCAGGACGAAGCAAGATCAC
MAPK14	F:AACAGGATGCCAAGCCATGA R:GCATCTTCTCCAGCAAGTCG
MST1	F:ACCCGTGTCGATATTAAGAGAC R:GCTCATCTTCATCCGAGTTTTC
hsa-miR-214-3p	F:CCCACAGCAGGCACAGACA
mmu-miR-214-3p	F:GCCACAGCAGGCACAGACA
GAPDH	F:GAAAGCCTGCCGGTGACTAA R:AGGAAAAGCATCACCCGGAG
U6	F:CTCGCTTCGGCAGCACA R:AACGCTTCACGAATTTGCGT

## Data Availability

The data that support the findings of this study are available from the corresponding author upon reasonable request.
